# COMPARATIVE ANALYSIS OF OUTCOMES OF OPEN OR ENDOSCOPIC SPINAL CORD DECOMPRESSION: INTEGRATIVE LITERATURE REVIEW

**DOI:** 10.1590/1413-785220263404e300681

**Published:** 2026-07-17

**Authors:** Victor Lima Dantas, Rafael Quixabeira Bezerra de Araújo, Taynan Siqueira Massaro, Abraão Lucas Dias Clerot Muniz Paiva

**Affiliations:** 1 Hospital Santa Marcelina de Itaquera, Serviço de Ortopedia e Traumatologia, Sao Paulo, SP, Brazil.

**Keywords:** Minimally Invasive Surgical Procedures, Low Back Pain, Recovery of Function, Intraoperative Complications, Procedimentos Cirúrgicos Minimamente Invasivos, Dor Lombar, Recuperação de Função Fisiológica, Complicações Intraoperatórias

## Abstract

**Introduction:**

Spinal cord compression is a neurological condition caused by pressure on the spinal cord, with etiologies including degenerative, neoplastic, infectious, traumatic, and malformative causes. It primarily affects the elderly, with symptoms ranging from pain and sensory-motor deficits to paralysis. The diagnosis is based on clinical evaluation and magnetic resonance imaging. Surgical treatment includes traditional open decompression and the endoscopic technique, the latter involving less trauma and quicker recovery.

**Objective:**

To review and compare the outcomes of spinal cord decompression via open and endoscopic approaches.

**Method:**

A search in the PUBMED database with the strategy: (decompre*[title] OR lamin*[title] OR discec*[title] OR micro*[title]) AND ((endosc*[title] OR minima*[title] OR percut*[title]) AND (open*[title] OR traditional[title])), with a 10-year time frame.

**Results:**

22 studies were identified; after reviewing titles and abstracts, 11 were excluded, leaving 11 articles that formed the sample.

**Conclusion:**

Minimally invasive techniques showed clinical efficacy comparable to open approaches in the treatment of lumbar disc herniation and lumbar spinal stenosis, with advantages in perioperative parameters. Variations in outcomes related to pain, function, complications, and reoperations suggest that the choice of technique should be individualized, considering the clinical presentation, the complexity of the lesion, and surgical expertise. ***Level of evidence II; review article.***

## RESUMO

**Introdução:**

A compressão medular é uma condição neurológica causada por pressão sobre a medula espinhal, com etiologias degenerativas, neoplásicas, infecciosas, traumáticas e malformativas. Afeta principalmente idosos, com sintomas variando de dor e déficit sensitivo-motor a paralisia. O diagnóstico baseia-se na avaliação clínica e na ressonância magnética. O tratamento cirúrgico inclui a descompressão aberta tradicional e a técnica endoscópica, esta com menor trauma e recuperação mais rápida.

**Objetivo:**

Revisar e comparar os desfechos da descompressão medular por via aberta e endoscópica.

**Método:**

Pesquisa na base PUBMED com a estratégia: (decompre*[title] OR lamin*[title] OR discec*[title] OR micro*[title]) AND ((endosc*[title] OR minima*[title] OR percut*[title]) AND (open*[title] OR traditional[title])), com corte temporal de 10 anos.

**Resultados:**

Foram identificados 22 estudos; após leitura de títulos e resumos, 11 foram excluídos, restando 11 artigos que compuseram a amostra.

**Conclusão:**

As técnicas minimamente invasivas apresentaram eficácia clínica comparável às abordagens abertas no tratamento da hérnia de disco lombar e da estenose espinhal lombar, com vantagens em parâmetros perioperatórios. Variações nos desfechos de dor, função, complicações e reoperações sugerem que a escolha técnica deve ser individualizada, considerando o quadro clínico, a complexidade da lesão e a expertise cirúrgica. ***Nível de evidência II; artigo de revisão.***

## INTRODUCTION

Spinal cord compression is a clinical condition characterized by pressure exerted on the spinal cord by adjacent structures, resulting in neurological impairment of varying degrees ^1^. This compression may have various etiologies, including degenerative diseases of the spine, such as spinal canal stenosis and large disc herniations; primary or metastatic neoplasms; infections such as epidural abscesses; trauma; and congenital malformations^2^. Furthermore, the progression of the condition may be acute, subacute, or chronic, depending on the underlying cause and the speed at which the compression occurs^1,2^.

Spinal cord compression primarily affects elderly individuals due to the higher prevalence of degenerative pathologies of the spine in this age group. However, young adults may also be affected, especially in traumatic or tumoral contexts^2–4^. Studies indicate that symptomatic lumbar stenosis, for example, affects up to 30% of the population over 60 years of age^2,5,6^. Regarding the distribution by sex, some types of compression, such as traumatic ones, have a higher prevalence in men, while neoplastic and degenerative causes are more equitable^4,5^.

The clinical consequences of spinal cord compression vary according to the level and severity of the injury, which may include intense pain, loss of muscle strength, impairment of tactile and proprioceptive sensitivity, alterations in sphincter control, and autonomic dysfunctions^1,7^. In severe cases, there may be partial or complete paralysis of the limbs below the level of the injury, directly impacting the patient's mobility and functional independence, as well as impairing their quality of life^2,7^.

The diagnosis of spinal cord compression is based on the correlation between clinical data and imaging findings^8^. Additionally, a detailed neurological evaluation is essential, including a physical examination and investigation of motor, sensory, and autonomic signs^1^. Magnetic resonance imaging (MRI) of the spine is the primary imaging study used, allowing for precise identification of the level, extent, and cause of the compression. In specific situations, computed tomography (CT) and myelography may also be employed as complementary examinations^8^.

The treatment of spinal cord compression may be conservative or surgical, depending on the etiology, severity of symptoms, and clinical condition of the patient^1^. Conservative measures include the use of analgesics, anti-inflammatories, physical therapy, and motor rehabilitation. However, in cases where there is progressive neurological deficit, intractable pain, or failure of clinical treatment, surgical intervention for spinal decompression becomes necessary, which may be performed through different access routes and surgical techniques^7,9^.

Open spinal decompression is a traditional technique that involves direct access to the spine through a wide incision, with dissection of the paravertebral tissues and exposure of the spinal canal^10^. Procedures such as laminectomy, foraminotomy, or discectomy are performed to remove the structures causing compression, such as bone fragments or disc herniations^2,11^. Although effective, this approach is associated with greater tissue aggressiveness, longer recovery time, and risk of perioperative complications^9,11^.

Endoscopic spinal decompression is a minimally invasive technique that allows for the removal of compressive elements through small incisions and the use of optical systems and specialized instruments^9^. This approach reduces trauma to soft tissues, preserves adjacent anatomical structures, and results in less blood loss, shorter hospitalization, and faster functional recovery, having gained traction as an effective alternative to the conventional technique in selected cases^2,11^.

In light of the increasing adoption of minimally invasive approaches in spinal surgery, it becomes relevant to comparatively evaluate the outcomes of open and endoscopic spinal decompression techniques. Thus, this work focuses on conducting an integrative literature review to identify which of the surgical modalities presents better results in terms of efficacy, safety, and functional recovery, contributing to evidence-based clinical decision-making.

## OBJECTIVE

To review the literature regarding the outcomes of spinal decompression via open or endoscopic methods and to compare them.

## METHOD

This exploratory work was developed through an integrative literature review, aiming to synthesize the available evidence. The search was conducted in the PUBMED database, utilizing the following search strategy: (decompre*[title] OR lamin*[title] OR discec*[title] OR micro*[title]) AND ((endosc*[title] OR minima*[title] OR percut*[title]) AND (open*[title] OR traditional[title])), considering a temporal cut-off of 10 years, from 2015 to 2025. Only clinical trials and observational studies were exclusively included.

The review was conducted according to the methodological steps described by Souza et al.^12^, which comprise: (a) definition of the guiding question; (b) survey of the available scientific production; (c) initial screening of the selected studies; (d) critical evaluation of the articles by experts; (e) analysis and interpretation of the findings; and (f) integration of the similarities and divergences identified among the examined studies. The guiding question used was: "what are the comparative clinical outcomes between spinal decompression performed via open and endoscopic methods, according to the evidence available in the scientific literature of the last 10 years?"

## RESULTS

Initially, 22 studies were identified that met the previously established search strategy. After reading the titles and abstracts, 11 articles were excluded for not directly addressing the outcomes of spinal decompression via open or endoscopic methods. The remaining 11 articles were read in full, summarized, and presented in the next section in chronological order considering the year of publication. Table 1 presents a summary of the main information related to the reviewed works.

**Table 1:** Summary of the main information related to the reviewed works.

Authors	Title	Type of Study	Number of patients	Conclusion
Choi et al. ^13^	*Percutaneous Endoscopic Lumbar Discectomy as an Alternative to Open Lumbar Microdiscectomy for Large Lumbar Disc Herniation*	Retrospective observational study	43	Percutaneous endoscopic lumbar discectomy (PELD) was effective in the treatment of lumbar disc herniation (LDH), with advantages such as reduced lumbar pain, preservation of disc height, faster recovery, and greater satisfaction compared to open lumbar microdiscectomy (OLM).
Cristante et al. ^14^	*Randomized clinical trial comparing lumbar percutaneous hydrodiscectomy with lumbar open microdiscectomy for the treatment of lumbar disc protrusions and herniations*	Randomized clinical trial	40	Hydrodiscectomy was as effective as open microdiscectomy, with similar rates of complications, recurrence, and satisfaction.
Chen et al. ^15^	*Traditional versus Percutaneous Transforaminal Endoforaminal Discectomy Effect on Nervous System Function and Serum LEK, GFAP, and PGE-2 in Patients with Senile Lumbar Spinal Stenosis*	Comparative experimental study	146	Percutaneous transforaminal endoscopic discectomy (PTED) proved superior to traditional surgery in terms of less blood loss, shorter hospitalization, and better neurological and lumbar function.
Ahn et al. ^16^	*Transforaminal Endoscopic Lumbar Discectomy Versus Open Lumbar Microdiscectomy: A Comparative Cohort Study with a 5-Year Follow-Up*	Prospective study with retrospective analysis	298	Transforaminal endoscopic lumbar discectomy (TELD) had clinical outcomes comparable to OLM, with advantages such as shorter surgical time, hospitalization, and return to work, despite the lack of randomization.
Song et al. ^17^	*Comparison of the Outcomes of Percutaneous Endoscopic Interlaminar Lumbar Discectomy and Open Lumbar Microdiscectomy at the L5-S1 Level*	Observational, retrospective, and matched cohort study	56	Percutaneous interlaminar endoscopic discectomy (PIED) had better perioperative results (less pain, shorter hospitalization and surgery time), although medium-term clinical and radiological outcomes were similar to those of OLM.
Gadjradj et al. ^18^	*Full endoscopic versus open discectomy for sciatica: randomized controlled non-inferiority trial*	Multicenter randomized clinical trial	488	PTED was not inferior to OLM in reducing leg pain and had slightly more favorable results in pain, function, and recovery, thus can be considered an effective alternative.
Quian et al. ^19^	*Transforaminal endoscopic lumbar discectomy using a 45° puncture angle and foraminotomy versus traditional THESYS for L5/S1 lumbar disc herniation: a prospective randomized controlled trial*	Randomized prospective clinical trial	Not Reported	The TELD at 45° was superior to traditional Thomas Hoogland spinal endoscopy systems (THESYS) in terms of operative time, pain (visual analog scale - VAS), Oswestry Disability Index (ODI), and lower complication rates; after three months, the outcomes were similar.
Baranidharan et al. ^20^	*24-Month Outcomes of Indirect Decompression Using a Minimally Invasive Interspinous Fixation Device versus Standard Open Direct Decompression for Lumbar Spinal Stenosis: A Prospective Comparison*	Randomized, prospective, multicenter clinical trial	48	The interspinous fixation device (IFD) proved to be safe and effective over 2 years, with significant improvement in pain, function, and less blood loss compared to surgical decompression.
Seddighi et al. ^21^	*Clinical Outcomes at 2-Year Follow-Up Comparing Open Surgery and Percutaneous Laser Disc Decompression for Radicular Sciatic Pain Patients*	Randomized and controlled prospective clinical trial	84	Percutaneous laser disc decompression (PLDD) and open surgery had similar efficacy in pain and function in the long term; PLDD had a higher reoperation rate but remains a viable alternative.
Hao et al. ^22^	*Clinical outcomes of unilateral biportal endoscopic discectomy (UBE) compared with conventional open lumbar discectomy with 3D microscope (OLDM) assisted*	Comparative and retrospective observational study	76	The unilateral biportal endoscopic technique (UBE) was comparable to open lumbar discectomy with a 3D microscope (OLDM) in clinical outcomes, but had advantages in shorter operative time, less blood loss, and shorter hospitalization.
Broekema et al. ^18^	*Percutaneous pedicle screw placement with a mini-open decompression versus open surgery in the treatment of lumbar spondylolisthesis: one-year results of a randomised controlled trial*	Multicenter randomized clinical trial	169	There was no significant difference between mini-open surgery and open surgery for spondylolisthesis; the expected advantages of mini-open surgery were not confirmed.

Source: Data collected by the authors.

## DISCUSSION

Choi et al.^13^, compared the results of lumbar disc herniation (LDH) treated with percutaneous endoscopic lumbar discectomy (PELD) and open lumbar microdiscectomy (OLM) through a retrospective observational study. The study was conducted from January 2011 to June 2012 with 44 consecutive patients diagnosed with LDH without cauda equina syndrome, scheduled for spinal surgery. Clinical outcomes were assessed using a visual analog scale (VAS), functional status was evaluated by the Oswestry Disability Index (ODI) at one, six, and 24 months postoperatively, in addition to the surgical satisfaction rate at the final follow-up. The radiological variables were evaluated through simple radiography. Forty-three patients were included; 20 and 23 patients underwent PELD and OLM, respectively. Both groups exhibited significant improvements in leg and back pain postoperatively, and although there was no significant difference in this regard between the groups, the improvement in back pain was significantly greater in the PELD group than in the OLM group. The surgical satisfaction rate of the PELD group was significantly higher than that of the OLM group, and the average operation time, hospital stay, and time until return to work were significantly shorter in the PELD group than in the OLM group. The height of the disc (%) significantly decreased from 23.7 ± 3.3 to 19.1 ± 3.7 after OLM, but did not change significantly after PELD (23.6 ± 3.2 to 23.4 ± 4.2). The segmental angle at the operated level increased from 10.3° to 15.4° in the PELD group, which was significantly greater than in the OLM group (9.6° to 11.6°). In the OLM group, there was one case of fusion due to instability, and in the PELD group, one case required revision surgery and another presented recurrence. There were no perioperative complications in either group. According to the authors, despite the small sample size and short follow-up period, it was possible to observe that PELD was an effective treatment for LDH and was associated with potential advantages, including rapid recovery, improvement of back pain, and preservation of disc height.

Cristante and collaborators^14^ evaluated the results of hydrodiscectomy compared to open microdiscectomy concerning pain, function, satisfaction, complications, and recurrence rates. To this end, they conducted a randomized clinical trial with patients referred to the authors' service for low back pain. Such subjects were included in the study if they presented disc protrusion or a small hernia at only one level, without neurological deficits and without resolution after six weeks of conservative treatment. One group underwent open microdiscectomy and the other group underwent percutaneous microdiscectomy by hydro-surgery. Function was assessed by the ODI and pain was assessed by a VAS. The evaluations were conducted preoperatively and subsequently during the first week and after one, three, six, and twelve months postoperatively. During the study period, 20 patients were included in each arm, and 39 completed one year of follow-up (one patient died from unrelated causes). Both groups showed equal improvement in the VAS and ODI assessments after treatment, with no significant differences. Furthermore, the improvement in the lumbar VAS score was not significant in the hydrodiscectomy group. The rates of infection, pain, recurrence, and satisfaction were similar between the two groups. According to the authors, percutaneous hydrodiscectomy proved to be as effective as open microdiscectomy in reducing pain. The rates of complications and recurrence of hernia were similar between the groups, and the patients' satisfaction with the treatment was also comparable.

Chen et al.^15^, compared the influence of percutaneous transforaminal endoscopic discectomy (PTED) and traditional surgery on the function of the nervous system and the serum levels of leuencephaline (LEK), glial fibrillary acidic protein (GFAP), and prostaglandin E-2 (PGE-2) in patients with senile lumbar spinal stenosis. To this end, they conducted an experimental study from March 2017 to March 2018 with 146 patients suffering from senile lumbar spinal stenosis from a single clinical center. The subjects were randomly divided into a control group and an observation group, with 73 in each group. The control group underwent traditional surgery, while the observation group underwent PTED. They compared the overall situation of the operation, serum LEK, GFAP, PGE-2, the score of the *American Spinal Injury Association* (ASIA), and the score of the *Japanese Orthopaedic Association* (JOA). The intraoperative blood loss in the observation group was less than in the control group; moreover, both the operative time and the duration of hospitalization in the observation group were shorter than in the control group. After 24 hours post-operation, the serum levels of LEK and the ASIA score in the observation group were higher than in the control group, while the serum levels of GFAP and PGE-2 and the JOA score in the observation group were lower than in the control group. According to the authors, compared to traditional surgery, PTED presented the advantages of less intraoperative blood loss, shorter operative time, and reduced duration of hospitalization, among others. Furthermore, PTED would effectively reduce the serum expression of LEK, BFGF, and PGE-2 in patients, and drastically improve the function of the nervous system and lumbar function.

Ahn and collaborators^16^, demonstrated the clinical outcomes of transforaminal endoscopic lumbar discectomy (TELD) compared to those of open lumbar microdiscectomy (OLM). To this end, between January 2009 and September 2011, 335 consecutive patients with symptomatic herniated discs were treated with decompressive discectomy, either by TELD or by open microdiscectomy. The patients were prospectively entered into the clinical database, and their medical records were reviewed retrospectively. Data from 298 patients treated with decompressive discectomy, either by TELD or by OLM, were evaluated with a minimum follow-up period of five years. Among them, 146 patients were treated with TELD (TELD group) and the remaining 152 patients with open microdiscectomy (OLM group). Perioperative data and clinical outcomes were assessed using the Visual Analog Scale (VAS), Oswestry Disability Index (ODI), and modified Macnab criteria. The VAS and ODI improved significantly in both groups, with an excellent or good outcome rate of 88.36% and 87.5% in the TELD and OLM groups, respectively. The reoperation rate was 4.2% and 3.3% in the TELD and OLM groups, respectively, and there were no significant differences in clinical outcomes. However, the operative time, length of hospital stay, and time to return to work were significantly shorter in the TELD group. Regarding the limitations of the study, the authors noted that the selection of patients was not randomized; therefore, the risk of bias may be increased. Furthermore, the study lacked an analysis of the radiographic changes related to degenerative alteration over the long-term follow-up period. The researchers concluded that the long-term results of TELD for soft LDH were comparable to those of conventional open microdiscectomy. Moreover, the technique of selective endoscopic discectomy under local anesthesia offered the typical advantages of minimally invasive procedures, such as reduced operative time, hospital stay, and recovery time.

Song et al.^17^, compared the clinical, surgical, and radiological outcomes of patients with disc herniation at the L5-S1 level who underwent endoscopic percutaneous interlaminar discectomy (PEID) or OLM, performed by a single surgeon with beginner-level proficiency. To this end, they conducted a retrospective observational study with a matched cohort that included 56 patients who underwent discectomy at the L5-S1 level, with a minimum follow-up of one year, between September 2012 and August 2016. The patients were allocated into two groups: a PEID group (n = 27; September 2014 to August 2016) and an OLM group (n = 29; September 2012 to August 2014). The clinical, surgical, and radiological outcomes were evaluated retrospectively. The baseline characteristics, including age, sex, medical history, body mass index, preoperative symptoms, and preoperative radiological findings, did not differ significantly between the groups. Furthermore, the overall clinical outcomes, including back and leg pain; the surgical outcomes, including blood loss, complication rate, and recurrence rate; and the radiological results, including degree of decompression, disc height, and sagittal alignment, were not significantly different between the two groups. However, the PEID group demonstrated significant advantages, including less immediate postoperative low back pain, favorable immediate postoperative Odom criteria, shorter operation time, shorter hospital stay, and a quicker return to work. According to the authors, their findings indicated that the PEID group achieved better perioperative outcomes, despite no significant difference between the groups in clinical and radiological results at mid-term follow-up.

Gadjradj and collaborators^18^, assessed whether DET was non-inferior to OLM in reducing leg pain caused by LDH. To this end, they conducted a multicenter randomized clinical trial with a non-inferiority design in four hospitals involving 613 patients aged between 18 and 70 years with at least six weeks of radiating leg pain caused by lumbar disc herniation. The study included a predetermined set of 125 patients who underwent DET, which were the cases with a learning curve performed by surgeons who had not performed DET prior to the study. Thus, the patients were separated into two groups, DET (n = 179) and OLM (n = 309). The primary outcome was self-reported leg pain, measured by a VAS from 0 to 100 at 12 months, assuming a non-inferiority margin of 5.0. The secondary outcomes included complications, reoperations, self-reported functional status, measured by the ODI, VAS for back pain, health-related quality of life, and self-perceived recovery. The outcomes were measured up to one year after surgery and analyzed longitudinally according to the intention-to-treat principle, and patients belonging to the DET learning curve were omitted from the primary analyses. At 12 months, patients randomized to DET reported a significantly lower VAS score for leg pain compared to patients randomized to open microdiscectomy. Blood loss was lower, the duration of hospital stay was shorter, and the timing of postoperative mobilization was earlier in the DET group than in the OLM group. The secondary outcomes reported by the patients, such as the ODI, the VAS for back pain, health-related quality of life, and self-perceived recovery, were similarly favorable to the DET. In one year, nine (5%) in the DET group, compared to 14 (6%) in the OLM group, underwent repeat surgery. The per-protocol analysis and sensitivity analyses, including patients from the learning curve, resulted in outcomes similar to the primary analysis. According to the authors, the DET was not inferior to open microdiscectomy in reducing leg pain. The DET showed more favorable results for self-reported leg pain, back pain, functional status, quality of life, and recovery. These differences, however, were small and may not reach clinical relevance; nevertheless, the DET could be considered an effective alternative to OLM in the treatment of sciatica pain.

Qian et al.^19^, conducted a prospective comparison of the efficacy and safety of TELD with a puncture angle of 45° *versus* the traditional Thomas Hoogland spinal endoscopy systems (THESYS) for the surgical treatment of LDH at L5/S1. To this end, consecutive patients with LDH L5/S1 undergoing TELD were randomized (1:1) and allocated to the TELD at 45° or THESYS groups. Clinical outcomes were assessed preoperatively, on the first day, and at three and six months post-surgery until the final follow-up. Surgical-related parameters, VAS scores, ODI, and modified MacNab criteria, as well as surgical complications, were recorded and analyzed. All patients were followed for at least 24 months, and compared to the THESYS group, the TELD at 45° group exhibited shorter operative time and intraoperative radiation time, as well as lower VAS scores for lumbar pain and leg pain during the intraoperative period. The VAS and ODI in the TELD at 45° group were significantly better than in the THESYS group at three months postoperatively. However, from three months onward, both groups exhibited comparable VAS and ODI scores. There was no significant difference between the two groups according to the modified MacNab criteria. There were two cases of residual disc and two cases of recurrence that required reoperation in the THESYS group. According to the authors, in cases of LDH L5/S1, the TELD technique at 45° was superior to the traditional THESYS in terms of surgical-related parameters and faster improvement in VAS and ODI, with a lower complication rate.

Baranidharan and collaborators^20^, conducted an early-stage, multicenter, randomized clinical trial with a five-year follow-up comparing the efficacy of a minimally invasive interspinous fixation device (IFD), implanted laterally, for direct open surgical decompression in the treatment of lumbar spinal stenosis (LSS). The results of the two-year study were presented for 48 participants randomly assigned to IFD or decompression. The primary outcomes of the study included changes from baseline at the eight-week, six, 12, and 24-month follow-ups for leg pain via VAS, back pain (VAS), ODI, physical function of LSS (via Zurich Claudication Questionnaire), distance walked in five minutes, and the number of repetitions from sitting to standing in one minute. The secondary outcomes of the study included the global impression of the patient and physician regarding change, adverse events, reoperations, operative parameters, and fusion rate. Both treatment groups demonstrated statistically significant improvements in average leg pain, back pain, ODI, physical function of the ESL, distance walked, and repetitions seated and standing compared to baseline over 24 months. The average reduction in ODI compared to baseline levels was between 35% and 56% for IFD and 49% to 55% for decompression at all follow-up time points. The average reduction in leg pain for the IFD group was between 57% and 78% for all time points, with 72% to 94% of participants experiencing at least a 30% reduction in leg pain from eight weeks to 24 months. The distance walked for the IFD group increased from 66% to 94%, and the repetitions seated and standing increased from 44% to 64% for all follow-up time points. Blood loss was 88% lower in the IFD group, and the operative time parameters strongly favored IFD compared to decompression. A fusion rate of 89% was assessed in a subset of IFD participants, and there were no issues with the intraoperative device or reoperations in the IFD group; only one consolidated and asymptomatic spinous process fracture was observed at 24 months. For the authors, despite the low number of participants in the IFD group, the study demonstrated successful clinical and safety outcomes over two years for IFD, with significant advantages related to the operation compared to surgical decompression.

Seddighi et al.^21^, compared the clinical outcomes of percutaneous laser disc decompression (PLDD) and open surgery in patients with radicular sciatic pain caused by LDH over a two-year follow-up period. To this end, they conducted a randomized controlled prospective clinical trial with 84 patients with chronic radicular pain allocated to the open surgery group (n = 42) or the PLDD group (n = 42). Patients were evaluated at the beginning of the study and at four, eight, 24, 48, and 96 weeks after the intervention. Outcome measures included the Roland-Morris Disability Questionnaire, the VAS for leg and back pain, and the body pain and physical functioning subscales of the *Short Form*-36. Reoperation rates were also recorded. No significant differences were observed in the Roland-Morris Disability Questionnaire scores, the VAS for leg and back pain, or the *Short Form*-36 between the two groups at any follow-up time point. Both groups showed improvement in disability and pain scores over time, with similar recovery patterns. The median reoperation rates were 19.0% for open surgery and 31.0% for PLDD (P = 0.314), indicating comparable long-term efficacy of both treatments. For the authors, the study demonstrated that PLDD and open surgery provide similar long-term outcomes in terms of disability, pain relief, and physical function for patients with radicular sciatic pain. They also noted that although PLDD was associated with a higher reoperation rate, it remained a viable minimally invasive alternative to open surgery. However, further research was necessary to refine patient selection criteria and improve the efficacy of the procedure for both interventions.

Hao and collaborators^22^, evaluated and compared perioperative parameters and clinical outcomes, including operative time, intraoperative blood loss, pain and modification, hospital stay, patient satisfaction, and complications, between open lumbar discectomy with a 3D microscope (OLDM) and the unilateral biportal endoscopic technique (UBE) for LDH. A total of 76 patients with LDH were included in this study from February 2019 to February 2022. All of them had undergone spinal surgery and were subjected to OLDM (42 cases) and UBE (34 cases) in two distinct surgical centers. Respectively, all patients had level 1 lumbar disc herniation. The estimated blood loss, operation time, hospital stay duration, and patient complications were compared between the two groups. The VAS for back and leg pain, the ODI, and the modified MacNab criteria were tested before surgery and three days, three months, and 12 months after surgery. Compared to the OLDM group, the UBE group had a shorter operative time, less intraoperative blood loss, and a shorter hospital stay. Furthermore, the VAS and ODI scores showed a significant reduction in both groups after the operation. There was no significant difference in the VAS and ODI scores preoperatively and three days, three months, and 12 months after the operation between the two groups. Meanwhile, there was no significant difference in the operational conversion rate and complications between the two groups. According to the authors, the application of OLDM produced clinical results similar to UBE for the treatment of LDH, including pain control and patient satisfaction. However, UBE was associated with several advantages over OLDM in terms of surgical time, intraoperative blood loss, short-term postoperative pain relief, and postoperative hospitalization.

Finally, Broekema et al.^23^ compared the placement of percutaneous pedicle screws *versus* open screws in patients undergoing decompression of the mid-lumbar line due to symptomatic lumbar spondylolisthesis, focusing on short-term low back pain. To this end, they conducted a randomized clinical trial carried out in surgical centers between 2015 and 2020. Participants with spondylolytic or degenerative lumbar spondylolisthesis were randomized to receive percutaneous pedicle screw placement with mini-open decompression (mini-open surgery) or conventional open surgery with instrumented fusion (open surgery). The primary outcome was short-term low back pain after two weeks, measured by an VAS. Leg pain, disability, and quality of life were recorded at two and six weeks, three and six months, and one year. Surgical variables, including complications, were recorded, and analyses were performed on the intention-to-treat population. In total, 169 participants were included and randomized to mini-open surgery (n = 81) or standard open surgery (n = 88). No statistically or clinically significant differences were found between the groups in terms of primary or secondary outcomes, and the duration of surgery, blood loss, hospital stay, and complications were also similar between the groups. According to the authors, the study did not detect a difference in outcomes between mini-open surgery compared to open surgery in patients with spondylolisthesis. Thus, the hypothetical advantage of short-term reduction of lower back pain, less blood loss, and better clinical outcomes could not be confirmed.

The analyzed studies largely agree on the clinical efficacy of various minimally invasive techniques for the treatment of lumbar disc herniation and spinal stenosis when compared to traditional open surgical approaches. Most studies reported significant improvement in VAS, ODI, and patient satisfaction in both groups, with few relevant clinical differences in the long term. There is consensus that endoscopic or percutaneous techniques, such as PELD, TELD, DET, PEID, hydrodiscectomy, and UBE, provided perioperative advantages, including shorter surgical time, less blood loss, shorter hospitalization, and quicker return to activities, as highlighted in studies such as those by Choi et al.^13^, Ahn et al.^16^, Song et al.^17^, Hao et al.^22^, and Gadjradj et al.^18^. However, some discrepancies emerge: while some studies, such as that by Cristante et al.^14^, did not observe significant superiority of the minimally invasive technique over open microdiscectomy in terms of pain and function, others, such as that by Chen et al.^15^, reported additional neurophysiological benefits with endoscopic techniques. There was also variation in complication and reoperation rates, with studies such as that by Seddighi et al.^21^, suggesting a higher reoperation rate with percutaneous techniques, although without significant clinical impact. Finally, Broekema et al.^23^, e Baranidharan et al.^20^, drew attention to the absence of clear clinical superiority between open and minimally invasive techniques in specific cases, emphasizing the importance of appropriate patient selection. Thus, despite the predominance of operative advantages of minimally invasive techniques, the findings are not uniform across all clinical contexts, and the choice of method should consider factors such as the type and level of the lesion, the surgeon's experience, and the patient's profile.

## CONCLUSION

The analyzed studies indicated that minimally invasive techniques, such as endoscopic and percutaneous discectomies, demonstrated clinical efficacy comparable to open surgical approaches in the treatment of lumbar disc herniation and lumbar spinal stenosis, with consistent advantages in perioperative parameters. However, the evidence also demonstrated variations in outcomes related to pain, function, complication rates, and reoperations, suggesting that the choice of the most appropriate technique should be individualized, considering the clinical picture, the complexity of the lesion, and the expertise of the surgical team.

## Data Availability

the underlying contents of the research text are contained in the manuscript.
